# An Extra Dimension to Decision-Making in Animals: The Three-way Trade-off between Speed, Effort per-Unit-Time and Accuracy

**DOI:** 10.1371/journal.pcbi.1003937

**Published:** 2014-12-18

**Authors:** Adrian J. de Froment, Daniel I. Rubenstein, Simon A. Levin

**Affiliations:** Department of Ecology and Evolutionary Biology, Princeton University, Princeton, New Jersey, United States of America; University of Washington, United States of America

## Abstract

The standard view in biology is that all animals, from bumblebees to human beings, face a trade-off between speed and accuracy as they search for resources and mates, and attempt to avoid predators. For example, the more time a forager spends out of cover gathering information about potential food sources the more likely it is to make accurate decisions about which sources are most rewarding. However, when the cost of time spent out of cover rises (e.g. in the presence of a predator) the optimal strategy is for the forager to spend less time gathering information and to accept a corresponding decline in the accuracy of its decisions. We suggest that this familiar picture is missing a crucial dimension: the amount of effort an animal expends on gathering information in each unit of time. This is important because an animal that can respond to changing time costs by modulating its level of effort per-unit-time does not have to accept the same decrease in accuracy that an animal limited to a simple speed-accuracy trade-off must bear in the same situation. Instead, it can direct additional effort towards (i) reducing the frequency of perceptual errors in the samples it gathers or (ii) increasing the number of samples it gathers per-unit-time. Both of these have the effect of allowing it to gather more accurate information within a given period of time. We use a modified version of a canonical model of decision-making (the sequential probability ratio test) to show that this ability to substitute effort for time confers a fitness advantage in the face of changing time costs. We predict that the ability to modulate effort levels will therefore be widespread in nature, and we lay out testable predictions that could be used to detect adaptive modulation of effort levels in laboratory and field studies. Our understanding of decision-making in all species, including our own, will be improved by this more ecologically-complete picture of the three-way tradeoff between time, effort per-unit-time and accuracy.

## Author Summary

Efficient decision-making is vital to the lives of all animals, but the underlying principles of how they achieve this are not yet fully understood. Researchers studying decision-making have generally assumed that animals balance a two-way trade-off between speed and accuracy: the more time they spend gathering information, the more accurate their decisions will be, but the greater the cost they have to pay. We suggest that this picture is missing a crucial component: the effort that animals spend on gathering information *within each unit of time*. This is important because an animal that can change the amount of effort it invests per-unit-time can use this ability to maintain the accuracy of its decisions even when it reduces the amount of time it spends on them, and can therefore gain a fitness advantage. We predict that this ability to change effort levels should therefore be widespread in nature. This updated view of a three-way trade-off between speed, effort per-unit-time and accuracy will help behavioral ecologists, neuroscientists, economists and psychologists to understand decision-making better, and may also lead to the development of more efficient control algorithms for robot decision-makers.

## Introduction

The conventional wisdom in behavioral ecology and neuroscience is that decision-making performance is the result of a tradeoff between speed and accuracy, and that animals balance this tradeoff according to the circumstances of each specific decision [1]. For instance, bumblebees foraging on artificial flowers sacrifice speed in favor of accuracy when the cost of picking the wrong flower type is increased [2] and *Temnothorax* ants deciding on a new nest-site sacrifice accuracy in the interests of speed as the urgency of their decision increases [3]. Evidence apparently consistent with such a tradeoff has been reported across a very wide range of systems and scales, including visual discrimination in rhesus macaques [4], olfactory discrimination in rats [5] and mice [6], predator avoidance in bumblebees [7], and nest site selection in honeybees [8].

We suggest that this picture is missing a crucial dimension: the fact that an animal can vary the amount of effort it expends *in each unit of time*. We use the term “effort per-unit-time” to refer to any investment an animal makes whose effect is to increase the amount of relevant information it acquires within each unit of time prior to making a decision. This includes expending resources on (i) sampling that information from the environment at a faster rate (for example by moving more quickly through the environment or by allocating more attention to the task [9]), and (ii) lowering the frequency of perceptual errors among those samples (for example by bringing the thermal and metabolic conditions of the sensory system closer to the optimum, or by investing in a more accurate sensory system on a developmental or evolutionary timescale).

Behavioral ecologists have long underlined the importance of the total expenditure of both time [10] and effort [11] in decision-making. However, the literature on speed-accuracy tradeoffs has focused on situations where the level of effort within each unit of time is fixed, and this has allowed for some simplifying assumptions such as assuming that time and effort are perfectly correlated, to the point that the terms “time” and “effort” are sometimes used interchangeably (in the absence of variable effort levels this makes sense, because total expenditure on effort increases linearly with time).

In nature, however, individuals can vary the amount of effort they invest in each unit of time. This breaks down the simplifying assumption of perfect effort-time correlation and requires new framework for studying how animals make decisions. The total effort invested in a task is the product of time and effort per-unit-time, and to a first approximation these can be varied independently of one another, though undoubtedly a higher effort per-unit-time will limit how long an animal can search. We do not consider this higher-order interaction in detail in this paper, although we do consider its implications in the discussion. Surprisingly there appears to have been no exploration of the simultaneous three-way trade-off involving the marginal costs of both time and effort.

### Time and effort per-unit-time are separate units of investment, and their relative cost will vary

In order to study this three-way tradeoff, we distinguish between the “baseline” cost of time, and the additional cost of the effort that is invested within each unit of time.

The “baseline” cost of each unit of time encompasses all those costs that accumulate at a fixed rate when an individual spends time on a particular activity, regardless of the level of effort per-unit-time that it devotes to that activity. These include, for example (i) the costs associated with predation risk (e.g. in each unit of time spent out of cover assessing potential food sources there is a particular probability that the individual will be spotted and then killed or injured by a predator, which will entail a fitness cost) and (ii) opportunity costs (e.g. each unit of time allocated to foraging cannot be spent searching for mates). The baseline cost of time might increase with the appearance of a predator (increasing the chance of being predated in each time unit spent out of cover), or of a potential mate (increasing the opportunity cost).

In contrast, the “cost of effort per-unit-time” is the cost to the animal of the resources *under its control* which it devotes to a particular task *within each unit of time*. These are costs over and above the “baseline” cost of time that are incurred as a result of spending additional energetic resources on a higher sampling rate or a lower error rate (as explained above). The cost of effort per-unit-time will therefore increase if a given unit of energetic expenditure becomes more costly in fitness terms, for instance because food is less abundant (and hence existing reserves are harder to replenish) or because the individual's energetic reserves are depleted (making the remaining reserves more valuable).

Effort per-unit-time and time are thus two different units of investment. Distinguishing between them is important. The baseline cost of time and the cost of effort per-unit-time will both vary depending on the states of both the animal and its environment. This variation is unlikely to be perfectly correlated: for instance, a change in an animal's food reserves does not perfectly predict the risk of predation, and the appearance of a predator does not perfectly predict the value of the animal's reserves. There will therefore be some fluctuation in the *relative* cost of time and effort.

### Substitution between time and effort per-unit-time should bring a fitness benefit

An animal that can modulate the amount of effort it invests within each unit of time can take advantage of this fluctuation in relative cost by substituting between time and effort as one becomes more expensive relative to the other. For instance when time is more expensive the animal can respond by spending less time on the task, but more effort within each unit of time that it does spend. It is thus “freed” from the constraints of the simple speed-accuracy trade off. It does not need to accept the same decline in accuracy that an animal facing that two-way tradeoff would face if it reduced its investment of time to the same degree, because it can increase the effort it expends in each of the remaining units of time, boosting its accuracy. This brings a fitness advantage. Similarly, when effort per-unit-time is more expensive the animal can respond by spending more time on the task, but investing less effort in each unit of that time, which should also bring a fitness advantage.

To take a hypothetical example, imagine an animal leaving its nest unguarded to gather a food item from one of two alternative patches. Let us assume that it gains a fitness benefit if it accurately chooses the richest patch. If a nest predator appears nearby, time spent foraging will become more expensive as the nest is more likely to be discovered and depredated in the parent's absence. The optimal strategy for an individual limited to a simple speed-accuracy trade-off will be to spend less time gathering information about the two patches and to return to the nest more quickly, accepting the reduction in accuracy that this will entail but improving the chance of preventing nest predation.

However, the appearance of the predator does not affect the marginal cost of effort, for instance the energetic cost of faster neural processing to reduce perceptual errors, or of faster movement between the patches to gather samples more quickly. Both of these factors could increase the amount of information the animal gathers about the patches in a given period of time. The optimal strategy for an animal that can modulate effort in this way will be to spend less time in assessing the two patches, but also to expend more effort in each of those units of time. It will thereby maintain better accuracy than an individual limited to a simple speed-accuracy trade off, and will therefore gain the fitness benefit of foraging from the richest patch more often.

### The model

In this paper we adapt a canonical model of statistical decision-making, the sequential probability ratio test (SPRT) [12], [13], to demonstrate that the ability to modulate effort levels does indeed confer a fitness advantage, and therefore that we should expect this ability to have evolved in nature. In the model, we examine effort of the second kind defined above: the investment an animal can make to reduce the perceptual errors that pollute the information it gathers before making a decision. Since both kinds of effort have the same effect of increasing the rate at which the individual gathers information, this choice does not affect the conclusions we can draw from our results and they apply equally to effort of the first kind (sampling rate).

Individuals in our model are tasked with making a binary choice about some (initially unknown) state of their environment, for instance whether a foraging patch is fruitful or not. They gather noisy evidence from their environment, and use the sequential probability ratio test (SPRT) [14] to make their decision.

The decision threshold an individual uses in the SPRT is defined by the value of the parameter 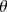 (increasing 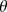 results in an increased investment of time spent gathering information, other things being equal). Its effort level is controlled by the parameter 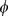 (increasing 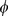 results in an increased investment of effort per-unit-time). See “Methods” for a fuller explanation of the operation of these. A unit of time spent gathering evidence imposes a fitness cost of 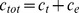, where 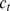 is the *baseline* cost per-unit-time of time spent gathering information and 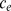 is the *additional* cost of effort per-unit-time. Making a correct decision brings a fitness benefit of 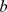 (measured in fitness units). We define the fitness of an individual as the benefit it obtains across all the decisions it makes, less the total cost it incurs in making those decisions (see equation (8) in “Methods”).

## Results

### The optimal values of the decision-making parameters depend on the relative costs of time and effort

**Figure 1:**
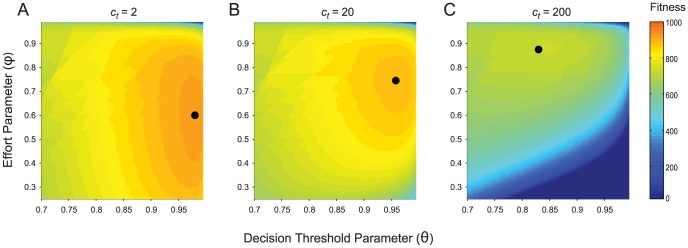
The optimal decision threshold and effort parameters for different values of the cost of time. Each point in these fitness landscapes shows the mean fitness of individuals using a particular pair of values of 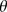, the decision threshold, and 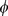, the effort parameter (which denotes the probability of making a perceptual error). We define fitness as net gain: deciding correctly brings a benefit, but the time and effort involved in each decision impose a cost (see Methods for details). The topology of the fitness landscape and the optimal values of 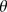 and *φ* (black circles) vary as we change the baseline cost of time, 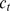. In panel **A** 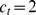, in panel **B** 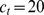 and in panel **C** 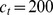. The optimum value of the decision threshold (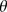) decreases as the cost of time rises, because individuals can no longer afford to collect so much evidence before making a decision. At the same time, the optimum value of the effort parameter (*φ*) increases, as individuals increase the investment they make to eliminate the perceptual errors in their sampling. Other parameter values: 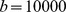, 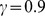, 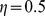, 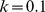, 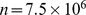 (see Methods for details).

We calculated the fitness of individuals (equation (8)) across a range of 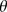 and 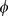 values, and examined how the optimum values moved as we changed *C_t_*, the baseline cost of time. All the fitness landscapes we examined had a single maximum point (e.g. Fig. 1) suggesting that there is a single optimum decision threshold, 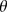, and effort level, 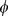, for each set of environmental parameter values. As we increased the cost of time, the optimal value of the decision parameter, 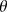 (which controls the decision time, other things being equal) decreased, while the optimal effort level, 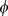, increased (Fig. 1a–c). This is because when the baseline cost of time is high, it pays to spend less time gathering evidence, but more effort ensuring that evidence that you do gather is error-free.

### Individuals that can control their investment of effort have higher fitness when relative costs change

**Figure 2:**
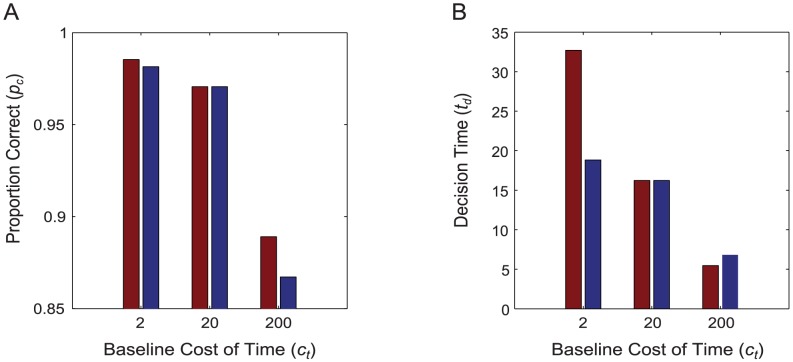
Comparison of individuals with and without the ability to modulate effort. Type 1 (red bars) can vary its level of effort (which it controls through the parameter 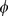) whereas type 2 (blue bars) cannot do so (it has a fixed value of 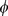). Type 2 has the value of 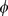 optimal for one particular value of the cost of time; in our example this is 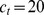. **A** Both types have the same performance under baseline regime of 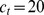, but when 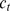 increases or decreases, type 1 makes a greater proportion of correct decisions than type 2. **B** If 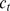 decreases, type 1 increases its decision time (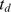) to a greater extent than type 2, and if 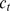 increases, it reduces its decision time to a lower level than the type 2. These results are from a very large number of simulations, so the error bars on these values are vanishingly small and all differences are significant. Other parameter levels: 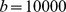, 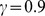, 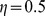, 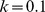, 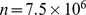 (see Methods for details and calculations).

**Figure 3:**
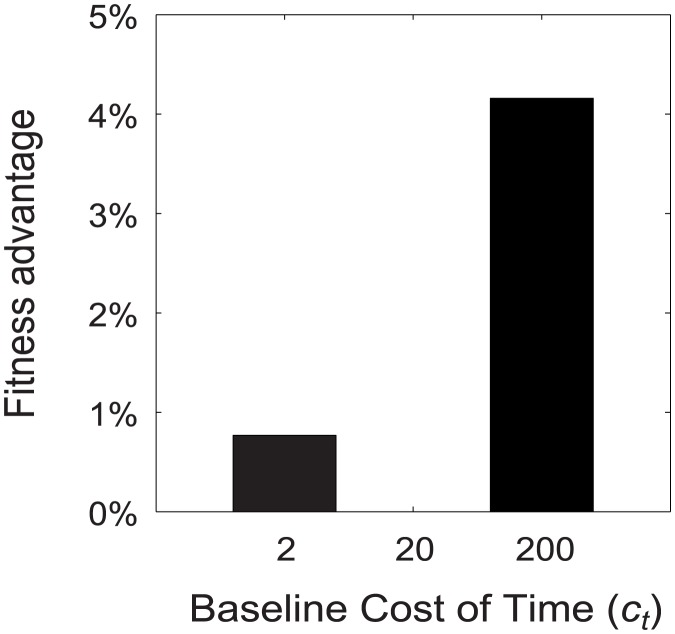
The fitness advantage of individuals who can modulate effort. When the cost of time (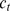) is raised or lowered from its baseline value, individuals of Type 1, who can control their level of effort, have a fitness advantage over individuals of Type 2, who cannot do so. Type 2 has an effort level adapted to the baseline regime under which it evolved (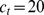). Therefore at 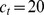, the fitness of the two types is exactly equal and the Relative Fitness Advantage is zero. Other parameter levels: 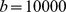, 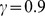, 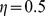, 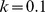, 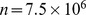 (see Methods for details).

**Figure 4:**
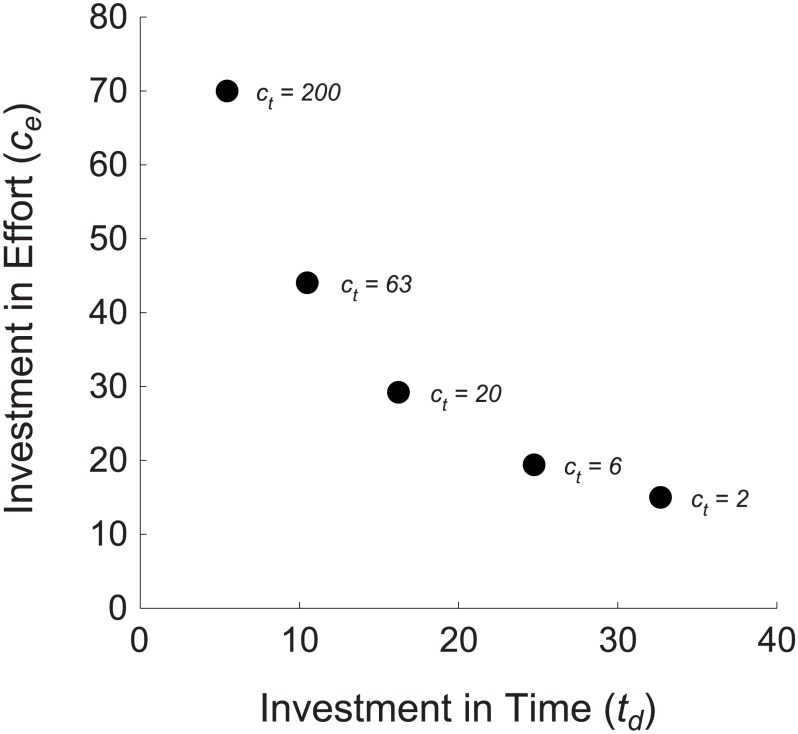
Effort and time are substituted for one another as their relative costs change. This figure shows our central result. The black circles show the mean expenditure of individuals of Type 1 on time and effort per-unit-time as the value of 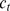 varies across two orders of magnitude. As the fixed costs of time (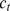) increase, optimally-adapted individuals substitute expenditure on effort per-unit-time (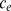), which is under their control, for expenditure on time (shown by the decrease in the total decision time 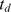). Where time is expensive, they spend more on effort and less on time, and where time is cheap, they spend more on time and less on effort in each unit of time. This effect is the behavioral manifestation of the changes in the underlying decision parameters 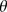 and 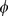, and could be measured experimentally. Other parameter levels: 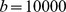, 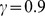, 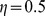, 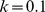, 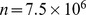 (see Methods for details).

Using these fitness landscapes, we then defined two groups of individuals. Type 1 could adapt both their threshold parameter 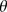 and their effort level 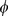 to the environmentally-determined optima, while Type 2 had a fixed effort level, with their value of 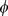 set to the environmentally-determined optimum for 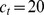. We computed the optimal values of the free decision parameters for different values of 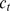, and compared the proportion of decisions that the two types made correctly (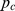), their mean decision time (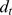) and their fitness (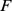). Individuals of Type 1 made more correct decisions than those of Type 2 at both 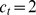 and 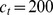 (Fig. 2a). They also increased their decision time more dramatically when the cost of time was decreased, and reduced it more when the cost was increased (Fig. 2b). They also had higher fitness (Fig. 3). They achieved this fitness gain because they substituted effort for time (or vice versa) as their relative costs changed. The central result that we seek to illustrate is shown in Fig. 4: as the baseline cost of time increases, individuals not only reduce their mean decision time, but also increase their mean expenditure on effort per-unit-time, 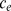. This option is not available to individuals with a fixed effort level, who cannot make the substitution between effort and time.

## Discussion

Our results provide a specific example that illustrates the broader conceptual point made in the introduction: an optimal decision-maker that can modulate the effort it invests per-unit-time will substitute effort for time when the relative cost of time increases, and this will give it a fitness advantage over individuals that cannot do so. We have shown that this effect is produced in the dynamics of the SPRT when the relative costs of time and effort are varied and individuals are allowed to vary their effort levels.

Investments of time and effort per-unit-time both lead to the same benefit: a greater number of accurate statistical samples and therefore a more accurate estimate of the state of the world and a more accurate decision. If the cost of a unit of time varies independently of the cost of a unit of effort, individuals who can substitute one currency for the other are able to exploit whichever one is cheapest. They will substitute effort for time when the baseline costs of time become relatively more expensive, and substitute time for effort when effort becomes more expensive (Fig. 4 shows this effect in the context of our model). In contrast, individuals limited to fixed effort level lack this flexibility. Because they cannot substitute into the cheaper currency, accuracy for them comes at a higher price. They therefore have lower fitness.

We hypothesize that control of effort per-unit-time will therefore be common in nature and suggest various avenues for experimental work on this topic (see Box 1 for our empirical predictions). It is notable that equivalent substitutions are well studied in other fields. For instance there is an interesting analogy between the speed-effort-accuracy tradeoff we suggest here and the theory of labor-capital tradeoffs in economics [15]. The role of variable effort in biology may have been overlooked until now simply because the cost of effort per-unit-time varies less in the laboratory than it does in animals' natural environments.

**Box 1. Empirical predictions**

***Behavioral Timescale***

We predict that levels of effort per-unit-time will vary on a behavioural timescale in systems including the following:

*Individual decisions*

1. **Mammalian brains**: changes in fMRI signals of neural activity reflect the energy consumption of different brain regions [20], and fluctuations in neural activity account for differences in response speed in decision tasks [21]. **Prediction**: fMRI signals in brain areas contributing to a decision will be stronger if the cost of time is increased.
2. **Bumblebees**: bumblebees can increase their temperature through non-shivering thermogenesis [2], [22]. Increased temperature is known to accelerate the response time of photoreceptors in other insects [23], and may offer a way for bumblebees to enhance their perceptual accuracy. **Prediction**: bumblebees foraging in low light will invest more energy in non-shivering thermogenesis when the cost of time is increased, for instance by increasing perceived predation risk.
3. **Human attention**: attention is a potentially useful metric of effort in humans. One common measure of how much attention human subjects are paying to decision tasks in the laboratory is how easily distracted they are. We expect attention levels, and thus also subjects' susceptibility to distraction, to vary in the circumstances we have predicted for effort more broadly. **Prediction**: human subjects who are given less time to complete a detection task will be less easily distracted by external stimuli, and those given a task that can be more efficiently solved using multiple senses will respond to distractions operating in fewer sensory modalities.

*Collective decisions*

4. **Eusocial insects**: the number of scouts sent out by honeybees [8] or *Temnothorax* ants [3] looking for a new nest site is a measure of effort at the colony level. The larger the number of colony members employed as scouts, the greater the opportunity cost the colony may have to pay in terms of missed foraging opportunities or colony maintenance tasks not done. **Prediction**: ant colonies will allocate more individuals to scouting when the cost of delay is increased, for instance by warming their old nest site so that their brood are in danger of desiccation.

*Social decisions*

5. **Animal contests**: in many species, animals decide the outcome of ritualized contests by a process of “mutual assessment” which is analogous to statistical decision making. They estimate one another's “strength” from information revealed by their aggressive interactions and the fight ends when one individual is sure enough that it is the weaker of the two that its optimal strategy is to stop fighting. Fights often escalate from cheap displays that reveal little information to more dangerous (and hence costly) physical interactions that reveal more information [24]–[26]. **Prediction**: individuals will escalate to the more informative physical interactions faster and fights will be decided sooner when the cost of time spent fighting is increased. For instance, contests between male zebrafish (*Danio rerio*) escalate from lateral displays to physical biting [27], and we predict that this escalation will occur more quickly in the presence of cues indicating the proximity of a predator.

***Evolutionary Timescale***

We also predict that variable levels of effort per-unit-time will be found on an evolutionary timescale, for example:

6. **Eyes**: Insects' compound eyes have excellent temporal but poor spatial resolution. They therefore integrate visual information over time in order to build up detailed spatial pictures [28]. In contrast, vertebrate eyes have excellent spatial resolution even with very short sample times but they require a much greater investment of developmental resources [29]. **Prediction**: species that occupy niches where time is more expensive will have eyes with better spatial resolution (for example by having more omatidia), controlling for phylogeny and other ecological factors that affect their visual needs.

We expect natural selection to favor the evolution of mechanisms to modulate effort per-unit-time when four conditions are met. First, we note that there is likely to be a baseline cost of maintaining the ability to modulate effort. This ability will therefore be favored whenever a species' environment varies enough that the benefits of the flexibility that ability brings outweigh this baseline cost. This general problem is analogous to others that are well understood. For instance, insect species with a distinct dispersal morph face a similar question: is environmental variability great enough that the benefits of developing dispersal ability outweigh the fixed costs involved (let alone the variable ones) [16]? We predict that species that occupy niches where there is relevant variation in the cost of time will be more likely to have evolved the ability to modulate their effort levels.

Second, the correlation between variation in the marginal baseline cost of a unit of time and variation in the marginal cost of a unit of effort per-unit-time must be less than 1. Where this is the case, optimally-adapted animals will be able to benefit by responding to rising time costs by substituting into effort and *vice versa*. In contrast, were the correlation perfect (equal to 1), the costs of time and effort could not vary relative to one another and so there can be no benefit from switching from one to the other. This is not the same as saying that the total expenditures of time and of effort are uncorrelated. They are obviously tightly correlated. This condition concerns variation in the costs per unit of time, not the total costs summed over time.

At present there appears to have been no explicit empirical investigation of the relationship between variation in the marginal cost of time and variation in the marginal cost of effort per-unit-time (Table 1 gives examples of these costs and possible sources of their variation). It seems likely that variation in the marginal costs of time will be only weakly correlated with variation in the marginal costs of effort. There is no literature that suggests, for example, that an increase in the probability of being discovered by a predator in any given unit of time predicts the cost of a unit of energetic expenditure. Of course the presence of a predator is likely lead to a greater total expenditure of energy (e.g. through fleeing or deterrence) but this is not the same as suggesting that it would result in an increase or decrease in the fitness cost of spending a single unit of energy. In any case, even if there is a non-zero correlation between changes in the cost of time and the cost of effort per-unit-time, there will be a fitness benefit to substituting between time and effort per-unit-time wherever that correlation is less than perfect.

**Table 1:** Marginal costs of time and effort per-unit-time and possible sources of variation.

	*Example of marginal cost*	*Sources of variation in that cost*
***Time***	The additional risk in each unit of time of being discovered by a predator and killed or injured.	Presence or absence of the predator nearby.
	The opportunity cost of not engaging in other fitness enhancing activities, for instance finding or attracting mates.	Presence or absence of a potential mate nearby.
***Effort per-unit-time***	The energetic costs of more acute sensory apparatus and/or faster neural processing (to process samples more accurately and quickly).	The marginal cost of expending energy will vary depending on the availability of food or oxygen from the environment, and the status of the animal's internal energy reserves.
	The energetic costs of faster physical movement (to gather samples more quickly).	

Third, there must be diminishing marginal returns to increasing investment in effort per-unit-time [17]. Diminishing marginal returns are ubiquitous in disciplines concerned with individuals' investment decisions such as biology and economics and would obviously be expected here. However it will be useful to test the magnitude of these diminishing returns explicitly in the case of effort per-unit-time, in order to verify that the relationship between cost and error is concave over the behaviorally relevant range and to parameterize our model.

Fourth, the fraction of the sampling error that can be eliminated by increasing effort per-unit-time must be sufficiently large. The noise in the samples an animal gathers is due both to the intrinsic variability of the environment and to the error introduced by its perceptual systems. If the first source of error swamps the second, the potential gains from increasing effort per-unit-time may not outweigh the costs, in which case we would not expect animals to vary their effort levels. Again, the relative magnitudes of these sources of noise have not yet been measured, and this is an interesting area for future work. We predict that species found in environments in which the signals they use for decision-making are noisier will be less likely to have evolved the ability to vary their effort levels. This condition would obviously not apply where the animal increases its effort by gathering more samples per unit time, rather than by reducing the errors in those samples.

Decision-making is central to the ability of all animals to survive and reproduce. The current view in behavioral ecology and neuroscience is that animals face a two-way tradeoff between speed and accuracy [1], [2], [18]. We suggest that effort per-unit-time, previously neglected, should be added as a third dimension in this tradeoff. If animals can adjust not only how long they spend gathering information prior to making a decision but also the effort they invest on gathering that information within each of those units of time, this can lead to an increase in both their accuracy and their fitness in the face of a changing environment. Current empirical evidence of the link between speed and accuracy in a wide range of species is consistent with this updated paradigm [3]–[8]. However, additional experiments comparing wild-type individuals with others that have had the hypothesized mechanisms of effort modulation “knocked out” would help to determine whether animals adaptively modulate their effort levels. Testing the hypothesis that animals have evolved to balance a three-way trade-off among speed, effort per-unit-time and accuracy will deepen our understanding of decision-making in all species, including our own, and may lead to the development of more efficient control algorithms for artificial decision-makers.

## Methods

### The sequential probability ratio test

In our model, individuals use the sequential probability ratio test to choose between two hypotheses about their environment [12].

We use a standard choice task in which an individual must decide whether some object is in state 1 or state 0. In order to inform this decision, the individual gathers a string of samples from the object. These samples are themselves either 1 s or 0 s with a probability that depends on the state of the object. The task here is to determine which of two known distributions the evidence is drawn from. This allows us to isolate the speed-effort-accuracy tradeoff from other factors. A more complex decision task that could also be used in this context is the multi-armed bandit problem. There, an individual is faced with *K* levers each providing a different (unknown) distribution of rewards when pulled, and its goal is to maximize the overall payoff. In the bandit problem the speed-effort-accuracy tradeoff is overlain with a second tradeoff between exploration (trying different arms) and exploitation (sticking with the arm believed to give the best payoff) [19].

In order make this abstract choice task easier to explain, let us imagine it within an imaginary ecological situation: individuals are foraging in a season where trees may be one of two types, good or bad (i.e. 1 or 0). They can gather evidence regarding the state of a given tree by examining the fruit husks lying beneath it. An individual will benefit if it can correctly decide whether a tree is good or bad before climbing up to the canopy to forage, but there is a cost to spending time and effort examining husks.

Of the fruit husks lying below a good tree a proportion 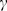 are a deep green color, indicating that they are fine, and contained nutritious material. The others are paler and drier, indicating that the fruit inside was rotten. Under a bad tree, a proportion 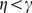 of the husks are from good fruit. Individual animals know the values of 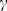 and 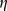, but not the state of the tree in front of them (which in our simulations is good 50% of the time and bad 50% of the time, chosen at random). Their task is to decide the tree's type.

Each husk therefore constitutes a piece of evidence that the animal gathers, 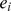, that has value 1 if the husk is 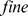 and 0 if it is 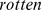. The individuals then apply the sequential probability ratio test to the evidence. Each piece of evidence is converted into a weight 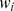, which is the log-likelihood ratio in favor of the hypothesis that the tree is good (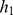) versus bad (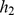). If the husk was observed to be fine, then the weight of evidence is given by
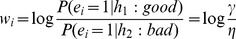
(1)and if it was observed to be rotten, then the weight of evidence is given by
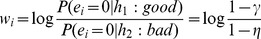
(2)These weights are summed over time to form the individual's decision variable. We measure time in terms of the number of pieces of evidence observed. After 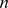 pieces of evidence, the decision variable is given by
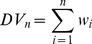
(3)The individual's decision thresholds are determined by the parameter 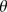. This parameter is bounded so that 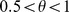. An individual decides that a tree is good if
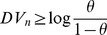
(4)and decides that the tree is bad if
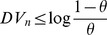
(5)The closer 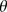 is to 1, the higher the threshold, and (with other parameters held constant) the more pieces of evidence the animal will gather before making a decision.

### Costs and benefits

Individuals make errors of perception, misjudging the state of a fraction of the husks they observe. The amount of effort they invest in assessing each husk is given by the parameter 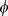. In the real world, effort might include the level of visual or olfactory attention used to examine a husk, the energetic cost of picking it up to assess its mass and the risks associated with tasting it. The value of 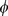 is the probability of correctly observing the state of a piece of evidence.

Each sample 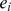 takes the true value of husk 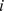 with probability 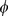, and with probability 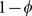 the value of 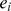 is chosen at random from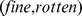. The value of 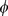 is bounded at 0 and 1. Individuals know their own level of error, and therefore use the adjusted parameters 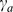 and 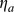 in the SPRT, where 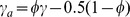 and 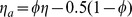. Acquiring each sample imposes a cost 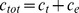, where 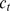 is the cost of the time taken to gather a sample and 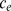 is the additional cost of the effort invested in ensuring that sample is accurate.

The value of 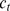 is determined by the individual's environment (so in our model we set it exogenously). In contrast, 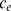 is a function of the amount of effort per-unit-time. We assume diminishing returns to increasing investment of effort and use a hyperbolic function for the relationship between 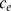 and 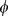. The probability of making an error-free observation, 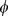, is given as a saturating function of 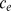:
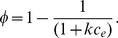
(6)where 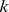 is a parameter that determines how quickly the reduction in error saturates with increasing expenditure on effort. This can be rewritten to give an expression for the cost 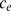 as a function of 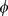:
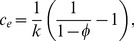
(7)In any given environment, the average cost an animal pays in order to reach a decision is therefore a function of the two evolving decision parameters, 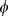 and 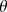. Between them, these affect the cost of examining each husk, and the total number of husks examined before a decision is reached.

Individuals gain a benefit 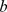 if the decision they make about the state of the tree they are under is correct.

### Calculation of optimal strategies

The optimal strategies were found approximately through use of a discrete grid of values for 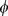 and 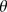. We simulated the mean decision time, 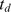, and the proportion of correct decisions, 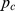, using an ensemble of 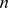 stochastic realizations of the model for each pair of values on this grid. We then calculated absolute fitness, 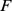, as
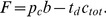
(8)The optimum strategy for a given set of environmental parameters is given by the pair of values of 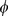 and 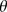 that led to the greatest fitness.

## Supporting Information

S1 Dataset
**Simulation code and output.** This dataset contains: (i) C++ code used to simulate 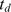
and 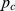 for a discrete grid of 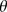 and 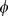 values; (ii) tab-delimited text files containing the output of those simulations; and (iii) matlab code used to calculate the optimum 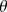 and 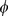 values for different levels of 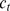 and to plot figs. 1–4.(ZIP)
